# Structural Characterization of *Neisseria gonorrhoeae* Bacterial Peroxidase—Insights into the Catalytic Cycle of Bacterial Peroxidases

**DOI:** 10.3390/ijms24076246

**Published:** 2023-03-26

**Authors:** Cláudia S. Nóbrega, Ana Luísa Carvalho, Maria João Romão, Sofia R. Pauleta

**Affiliations:** 1 Microbial Stress Lab, UCIBIO—Applied Molecular Biosciences Unit, Department of Chemistry, NOVA School of Science and Technology, Universidade NOVA de Lisboa, 2829-516 Caparica, Portugal; 2 Associate Laboratory i4HB—Institute for Health and Bioeconomy, NOVA School of Science and Technology, Universidade NOVA de Lisboa, 2829-516 Caparica, Portugal; 3 Macromolecular Crystallography Lab, UCIBIO—Applied Molecular Biosciences Unit, Department of Chemistry, NOVA School of Science and Technology, Universidade NOVA de Lisboa, 2829-516 Caparica, Portugal

**Keywords:** *Neisseria gonorrhoeae*, bacterial peroxidase, active state, azide-inhibited structure, ROS detoxification, catalytic cycle, diheme enzymes

## Abstract

*Neisseria gonorrhoeae* is an obligate human pathogenic bacterium responsible for gonorrhea, a sexually transmitted disease. The bacterial peroxidase, an enzyme present in the periplasm of this bacterium, detoxifies the cells against hydrogen peroxide and constitutes one of the primary defenses against exogenous and endogenous oxidative stress in this organism. The 38 kDa heterologously produced bacterial peroxidase was crystallized in the mixed-valence state, the active state, at pH 6.0, and the crystals were soaked with azide, producing the first azide-inhibited structure of this family of enzymes. The enzyme binds exogenous ligands such as cyanide and azide, which also inhibit the catalytic activity by coordinating the P heme iron, the active site, and competing with its substrate, hydrogen peroxide. The inhibition constants were estimated to be 0.4 ± 0.1 µM and 41 ± 5 mM for cyanide and azide, respectively. Imidazole also binds and inhibits the enzyme in a more complex mechanism by binding to P and E hemes, which changes the reduction potential of the latest heme. Based on the structures now reported, the catalytic cycle of bacterial peroxidases is revisited. The inhibition studies and the crystal structure of the inhibited enzyme comprise the first platform to search and develop inhibitors that target this enzyme as a possible new strategy against *N. gonorrhoeae*.

## 1. Introduction

Gonorrhea is a sexually transmitted disease caused by the obligate human pathogen *Neisseria gonorrhoeae.* According to the Centers for Disease Control and Prevention (www.cdc.gov (accessed on 2 February 2023)) *N. gonorrhoeae* represents an urgent threat because of its high drug resistance and the potential to become a serious worldwide threat. There are no vaccines against this microorganism, and since it progressively develops resistance to antibiotics, research on new targets has become critical.

To survive in the host, pathogenic bacteria need to cope with reactive oxygen species (ROS) originating from extracellular (host immune system and other surrounding microorganisms, such as *Lactobacillus* sp.) and intracellular sources. *N. gonorrhoeae* has many mechanisms to detoxify ROS [1], and for removing H_2_O_2_ in particular it uses two enzymes: a catalase (KatA) [2], located in the cytoplasm, and a bacterial peroxidase (*Ng*BCCP) [3], located in the periplasm.

Bacterial peroxidases (commonly named bacterial cytochrome *c* peroxidases) (BCCPs) reduce hydrogen peroxide to water and are widespread among Gram-negative bacteria, including other pathogenic bacteria, such as *Campylobacter jejuni* [4].

*Ng*BCCP was the first BCCP from a pathogenic bacterium to be biochemically and spectroscopically characterized [3,5], indicating that it plays a relevant role in the detoxification of hydrogen peroxide. This enzyme, unlike other classical BCCPs, is not soluble in the periplasm as it is anchored to the outer membrane by a lipid-modified cysteine. Between the N-terminus modified cysteine and the conserved globular domain there is a low complexity linker region, rich in alanine residues (imperfect repeats of AAEAP), which is also found in other gonococci proteins [6], such as LAz (lipid modified azurin, *Ng*BCCP electron donor [7,8]), Lip (lipoprotein), and AniA (nitrite reductase).

Furthermore, bioinformatic analysis of the *Ng*BCCP amino acid sequence and its biochemical characterization found that the globular domain is homologous to classical BCCPs, with a high-potential electron-transferring heme (E heme) His/Met 6-coordinated in the C-terminal domain and a low-potential peroxidatic heme (P heme) in the N-terminal domain, the active site [5]. In classical BCCPs, the P heme is bis-His 6-coordinated in the fully oxidized state, and upon reduction of the E heme (reductive activation) in the presence of calcium ions, the P heme loses its distal histidine ligand, gaining the ability to bind the substrate, hydrogen peroxide [9,10]. This reductive activation of classical BCCPs involves a series of conformational changes, mainly around the P heme [11,12,13]. In this process, the loop containing the distal axial P heme ligand moves away, and a π-stacking interaction at the homodimer interface is formed between the aromatic side chain of a tryptophan residue and the peptide bond of a glycine residue of the opposite chain (Trp87 and Gly72, with His85 being the distal axial P heme ligand, numbering according to *Paracoccus pantotrophus* BCCP [12]).

In common with the classical bacterial peroxidases, *Ng*BCCP requires reductive activation and the presence of calcium ions for maximum catalytic activity [5]. However, *Ng*BCCP differs from those enzymes as it only forms a dimer in the presence of calcium ions (with one high-affinity calcium binding site per monomer) and at high protein concentrations but not at high ionic strength, as reported for other classical BCCPs [9]. This was explained considering that the dimer interface of *Ng*BCCP could be less hydrophobic [5]. Spectroscopically, *Ng*BCCP’s P heme has also the unique feature of being high-spin at cryogenic temperatures, which has been ascribed to changes in the P heme cavity or geometry [5], leading to higher p*Ka* of the OH-group that coordinates the P heme in this redox state [14]. These exclusive properties can now be explored with the analysis of its crystal structure.

Because *Ng*BCCP is a highly conserved enzyme in *N. gonorrhoeae* strains, with a unique cellular location and absent from human cells, it is a good candidate to design molecules that inhibit this enzyme and that could constitute potential active compounds against *N. gonorrhoeae*. To inhibit *Ng*BCCP activity it is necessary to search for exogenous ligands that bind *c*-type cytochromes, and several examples in the literature could be explored [15,16,17]. Those ligands bind accessible hemes such as the 5-coordinated P heme and as a result they alter their properties and/or inhibit their catalytic activity. The binding affinities will depend on the heme binding pocket, protein ligands, and bond stability.

Spectroscopic studies with exogenous ligands (azide, cyanide, cyanate, carbon monoxide, fluoride, and imidazole) have been performed for BCCPs isolated from *Pseudomonas aeruginosa* [18], *P. pantotrophus* [19], *Rhodobacter capsulatus* [20], and *Nitrosomonas europaea* [21]. These compounds were shown to bind the mixed-valence state of BCCP, affecting the P heme spectroscopic properties of all these enzymes, and in the case of *N. europaea* BCCP these compounds also bound the fully oxidized state, as this enzyme does not require reductive activation [21]. The effect of azide and cyanide in the kinetic parameters was examined for *P. aeruginosa* and *N. europaea* BCCPs. For the former enzyme, azide and cyanide showed a non-competitive and mixed inhibition mechanism, respectively [18], while for the latter enzyme, the mechanism of inhibition was found to be mixed and competitive for these two compounds, respectively [22].

The *Ng*BCCP three-dimensional structure in the active and inhibited forms, as well as the inhibition studies presented here, provides the first insight into compounds that can be developed toward *N. gonorrhoeae* targeting *Ng*BCCP. Furthermore, the analysis of these structures, especially of the active site (the P heme), sheds light on the P heme spectroscopic properties, given that *Ng*BCCP displays a dynamic monomer/dimer equilibrium. The analysis of the heme pocket residues and of the catalytic properties of this enzyme was used to revisit the catalytic mechanism of BCCPs.

## 2. Results

### 2.1. Inhibition Studies—Binding and Steady-State Kinetics

**Figure 1:**
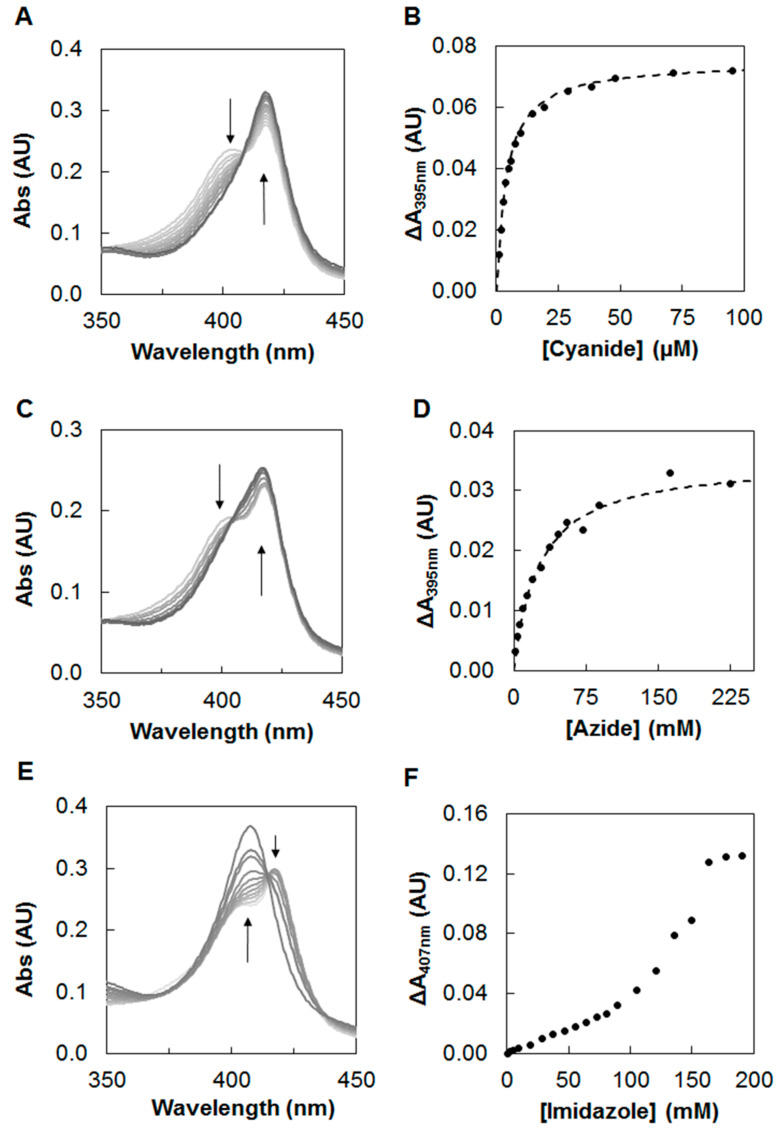
UV-visible spectra of mixed-valence *Ng*BCCP at pH 7.5 in the presence of increasing concentrations of cyanide (**A**), azide (**C**) and imidazole (**E**). The arrows indicate the direction of changes in the spectra from the light gray (no inhibitor) to dark gray (maximum inhibitor concentration). The absorption difference at specific wavelengths was plotted as a function of inhibitor concentration in solution ((**B**), cyanide; (**D**), azide; (**F**), imidazole). The dashed line (**B**,**D**) in the binding of cyanide and azide was fitted with a single binding site equation, with a *K_app_* of 4 µM and 26 mM, respectively.

To assess if the *Ng*BCCP active site (P heme) was available to bind exogenous ligands, three known heme-binding compounds were tested: cyanide, imidazole, and azide. The binding of these compounds was monitored by following the changes in the UV-visible spectrum of the mixed-valence *Ng*BCCP (active state) upon increasing concentrations of the inhibitor (Figure 1).

In the Soret region of this spectrum, there is a main absorption band at 420 nm corresponding to the reduced E heme and a shoulder at around 402 nm attributed to the oxidized P heme in a high-spin configuration [5,9,10]. The absorbance changes were monitored at 395 nm, a wavelength at which there is a maximum of absorbance change in the difference spectra (mixed-valence *Ng*BCCP in the absence minus in the presence of exogenous compounds). Moreover, a decrease in the absorbance at this wavelength, as well as in the 380 nm region, corresponded to a decrease in high-spin state of P heme [19].

Addition of cyanide or azide caused a decrease in the absorbance at 395 nm, which was consistent with a high- to low-spin transition at the P heme. The variation of absorbance at this wavelength as a function of inhibitor concentration was analyzed using Equation (1) (Section 4) considering a single binding site at the P heme. A *K_app_* for cyanide was estimated to be 4 µM, while azide bound with a *K_app_* of 26 mM (Figure 1C,D) at pH 7.5. However, at pH 6.0 the affinity was higher, as azide bound with a *K_app_* of 3.8 mM (see Figure S1 in Supplementary Materials).

The binding of imidazole had two phases. At lower concentrations of imidazole (0–50 mM), as observed by the increase at A_407 nm_, binding occurred mainly at the P heme (similar to cyanide and azide, there was also a decrease in absorbance in the 390 nm region), while at higher concentrations (>50 mM) there was a decrease at A_421 nm_ and an increase at A_410 nm_ as the Soret band of the reduced E heme was also affected (Figure 1E,F).

In order to determine how the binding of these inhibitors affected *Ng*BCCP activity, kinetic assays in the presence of various concentrations of the inhibitor and H_2_O_2_ were performed, using ABTS^2−^ an artificial electron donor [5] at saturating concentrations.

**Figure 2:**
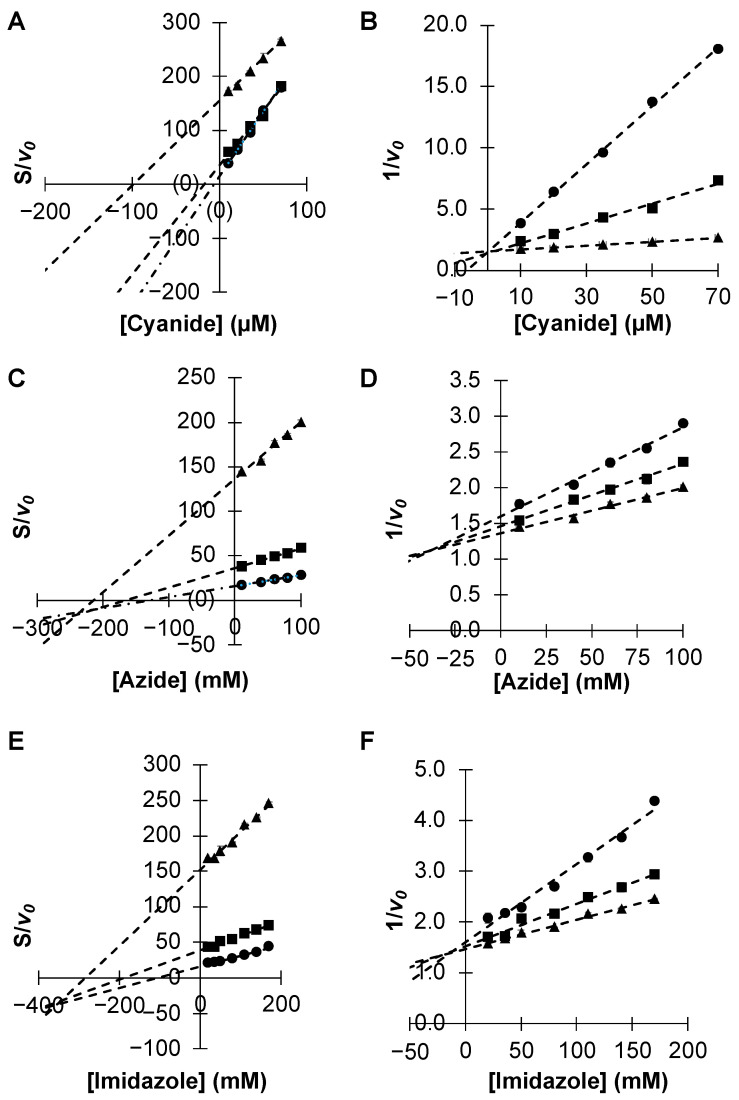
Determination of the inhibition constants for (**A**,**B**) cyanide, (**C**,**D**) azide, and (**E**,**F**) imidazole at pH 7.5. *K_i_’* and *K_i_* estimates are given by the intercept value in the plots of S/*v_0_* (Dixon: **A**,**C**,**E**) or 1/*v_0_* (Cornish–Bowden: **B**,**D**,**F**), respectively, against i at three different substrate concentrations (● 0.1 µM, ■ 25 µM, and ▲ 100 µM H_2_O_2_). The error bars represent the average of duplicate assays.

The *K_M.app_* estimated in the presence of cyanide was higher than *K_M_* (value in the absence of inhibitor), while the *V_M.app_ ≈ V_M_* (see Table S1 in Supplementary Materials). In the Dixon plot (Figure 2A) there was no intercept, and so no uncompetitive inhibition constant (*K_i_*’) [23] could be calculated. The mechanism of inhibition of cyanide was purely competitive (with the value of α being very large). The *x-*intercept in the Cornish–Bowden plot [24] gave the estimated −*K_i_* (Figure 2B), although an average *K_i_* of 0.4 ± 0.1 µM was calculated from the *K_M.app_* according to Equation (2). A non-linear fitting of both *K_M.app_* and *V_M.app_* gave a *K_i_* value of 2.3 ± 0.2 µM (see Figure S2 and Table S2 in Supplementary Materials).

**Scheme 1:**
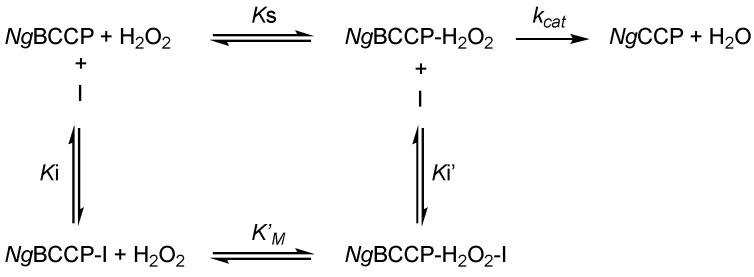
Kinetic diagram of *Ng*BCCP activity with H_2_O_2_ as substrate and H_2_O as final product (protons and electrons in this reaction were omitted for simplicity). In the presence of the inhibitor (I) there is a mixed inhibition, where *K_i_* is the binding constant to the free enzyme and *K_i_*’ the binding constant in the presence of substrate. These two constants are related by a constant α, with *K_i_*’ = α.*K_i_*, and *K*’*_M_* = α*Ks* (see Section 4).

The *K_M.app_* values estimated in the presence of azide and imidazole were also higher than *K_M_*, which meant that the apparent affinity of the enzyme to the substrate decreased in the presence of the inhibitor, favoring binding of the substrate to the free enzyme, as observed for cyanide. However, for these two inhibitors, *V_M.app_* < *V_M_*, which indicated that they also bound to an allosteric site. The data indicated that there was a mixture of competitive and uncompetitive binding as shown in the kinetic diagram in Scheme 1.

The constants *K_i_’* and *K_i_* were estimated from the Dixon (Figure 2C,E) and Cornish–Bowden (Figure 2D,F) linear plots for both azide and imidazole, in order to determine α. In the case of azide, *K_i_* of 41 ± 5 mM, *K_i_’* of 238 ± 4 mM, and α of 6.4 ± 0.4 were estimated at pH 7.5 from *V_M._*_app_, according to Equation (3) (Material and Methods). Imidazole showed a lower competitive inhibition constant, with *K_i_* of 9 ± 4 mM, *K_i_’* of 350 ± 5 mM, and *α* of 28 ± 5. In both cases, the value of *α* being above 1 indicated that the presence of the inhibitor decreased the affinity of the enzyme for the substrate, and since it was a linear type of inhibition, the binding of the inhibitor to the enzyme–substrate complex was non-productive (Scheme 1).

Contrary to what was observed in the titration with imidazole (Figure 1E,F), these plots do not show a different behavior below and above 50 mM (Figure 2E,F). However, a different representation of the data evidenced a different trend above 50 mM of imidazole (see Figure S2 in Supplementary Materials). The analysis of the complete range of imidazole concentrations indicated a non-competitive/partially competitive inhibition, similar to the previous analysis (see Figure S2 and Table S2 in Supplementary Materials), while until 50 mM imidazole the parameters that were estimated pointed to a partially uncompetitive inhibition (the value of α and β were identical, and α < 1, fitting not shown).

### 2.2. Crystallization of the Active and Azide Inhibited NgBCCP

Several of the crystallization conditions tested for the as-isolated *Ng*BCCP produced crystals, but these were not reproducible or showed multiplicity. Pre-incubating the enzyme with CaCl_2_ improved the reproducibility, as expected, since it reduces the enzyme solution states, making it a more homogeneous sample and increasing the enzyme’s stability [5].

Including additives in the condition containing Jeffamine SD2001 as precipitant led to single crystals. The additive 1,6-hexanediol promoted the formation of single plates and hexamine cobalt (III) trichloride led to tetragonal crystals. These conditions were extensively explored to optimize and produce better crystals, but all the crystals obtained using Jeffamine SD2001 as precipitant were fragile and diffracted poorly. The only other conditions that produced crystals of as-isolated *Ng*BCCP were the ones using 5/4 PO/OH as precipitant, which resulted in small and thin needle crystals that were, however, dependent on protein batch and thus not reproducible.

The crystallization conditions of the as-isolated *Ng*BCCP were then used in trials with the mixed-valence state. To have a constant anoxic environment, the drops were prepared inside an anaerobic chamber in the presence of a reducing agent (sodium ascorbate) and a redox mediator (flavin mononucleotide (FMN)) with a negative reduction potential (−205 mV at pH 7.0) at the working pH range (pH 5.5 to 6.5). The best crystals were obtained in 0.1 M MES pH 6.0, 30% 5/4 PO/OH, 2 mM CaCl_2_, 10 mM sodium ascorbate, and 0.2 mM FMN.

The size of the crystals varied from plate to plate, probably because of small variations in temperature during the preparation of the drops inside the anaerobic chamber prior to their incubation at 30 °C. The smaller crystals appeared in 26–30% 5/4 PO/OH, while the larger crystals only appeared in 30% 5/4 PO/OH.

### 2.3. Structural Analysis of the Active NgBCCP

Two structures of *Ng*BCCP in the mixed-valence state were solved, one to a final resolution of 1.8 Å acquired in-house and another of 1.4 Å using synchrotron radiation (SLS data). The in-house structure was solved by molecular replacement, using the mixed-valence structure of *P. aeruginosa* BCCP (PDB ID:2VHD) as a model (48% amino acid sequence identity). This preliminary in-house structure served as phases for the higher resolution synchrotron data. A third structure, the azide-inhibited *Ng*BCCP, was solved to a 2.3 Å resolution (ESRF data).

**Figure 3:**
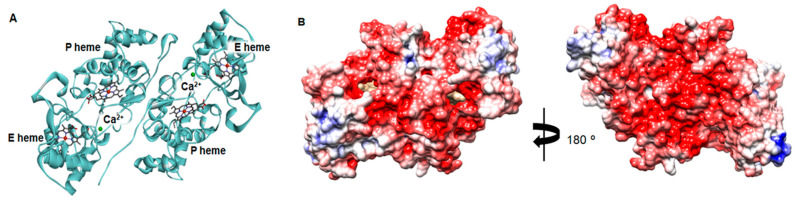
Structure of the mixed-valence *Ng*BCCP homodimer. In (**A**) is shown the dimer backbone, which comprises the asymmetric unit of the crystal, and in (**B**) its electrostatic surface potential is represented from −5 to 5 KT/e (colored from negative red surface to positive blue surface). Panel (**A**) was prepared with BIOVIA Discovery Studio Visualizer 4.5 and Panel (**B**) with Chimera 1.10.2.

The crystals belonged to the *P*2_1_2_1_2_1_ space group and the *Ng*BCCP dimer constituted the asymmetric unit. Each monomer had one calcium atom and was composed of two domains of cytochrome fold, each one harboring a *c-*type heme. The dimer structure is shown in Figure 3A and its surface is colored by electrostatic potential (Figure 3B). The C-terminal His-tag was solvent-exposed and not observed in any of the monomers because of mobility. Overall, the surface of *Ng*BCCP was negatively charged except at the E heme domain, which was more neutral with small positively charged patches (Figure 3B).

**Figure 4:**
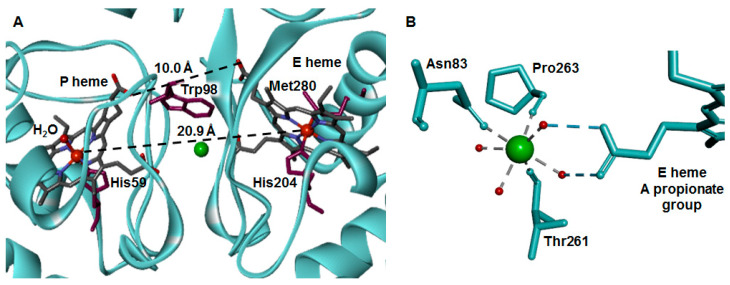
View of the mixed-valence *Ng*BCCP structure and calcium binding site. In (**A**) is shown the calcium ion (green sphere) located between the two hemes, the His/Met 6-coordinated E heme and the P heme coordinated by a proximal histidine residue and a distal water molecule (small red sphere). Although the two hemes have a Fe-Fe distance of 20.9 Å, electron transfer is possible because of the tryptophan residue located between their propionate groups. In (**B**) is shown the calcium binding site. The calcium ion (green sphere) is coordinated by the oxygens of four water molecules (small red spheres) and three conserved residues (Asn83, Thr261 and Pro263). This view also shows that the calcium ion is near the carboxylate group of the E heme propionate A, which forms hydrogen bonds with two of the coordinating water molecules. Figures prepared with BIOVIA Discovery Studio Visualizer 4.5.

The common features among the BCCPs are shown in Figure 4, which include the E and P hemes, their axial ligands, the coordination of the calcium binding site and the tryptophan residue (Trp98) between the two heme domains. In the mixed-valence *Ng*BCCP, the E heme was 6-coordinated by the conserved Met280 and His204 residues, and the P heme was 6-coordinated by His59 and a solvent-derived molecule. Similar coordination of the E and P hemes has been observed in other BCCPs in the mixed-valence state [11,12]. These two hemes were perpendicular to each other, with a Fe-Fe distance of 20 Å and between them lay the conserved Trp98 (Figure 4A), proposed to mediate the electron transfer between the two heme irons [12,20]. The calcium ion was coordinated by four water molecules and three conserved residues: the oxygen amide of Asn83 and the carbonyls of Thr261 and Pro263 (Figure 4B) in a pentagonal bipyramidal geometry, as first described in the *P. aeruginosa* BCCP crystal structure [25]. The calcium site was near the E heme A propionate group, which was hydrogen-bonded to two of the four water molecules (Figure 4B).

**Figure 5:**
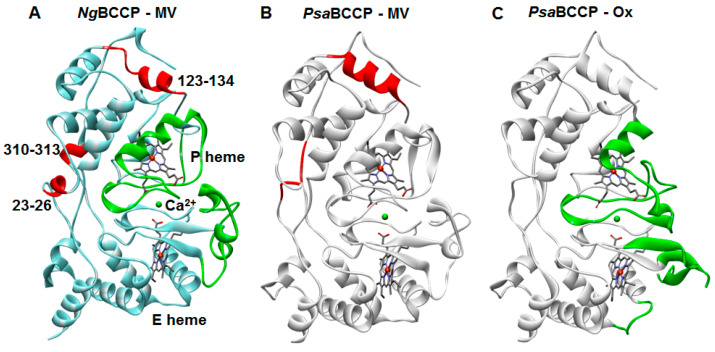
Structure changes that occur upon activation. Structure comparison of (**A**) *Ng*BCCP mixed-valence (MV) state with (**B**) *P. aeruginosa* BCCP in the MV (*Psa*BCCP–MV) and (**C**) oxidized (*Psa*BCCP–Ox) state. In red are highlighted the structural differences between MV states, and in green the differences between oxidation states. Figures prepared with BIOVIA Discovery Studio Visualizer 4.5 using PDB ID 2VHD and 1EB7 for *P. aeruginosa* BCCP in the mixed-valence and oxidized state, respectively.

The *Ng*BCCP structure was superimposed with all the available BCCP structures (see Table S3 in Supplementary Materials). Overall, the structures were similar, especially the mixed-valence structures, but *P. aeruginosa* BCCP (*Psa*BCCP) in the mixed-valence state (PDB ID: 2VHD) showed the lowest RMSD (1.16 Å) and was thus used to address any structural differences between the active states of *Ng*BCCP and *Psa*BCCP (Figure 5). In the absence of an oxidized *Ng*BCCP structure, the structure of the oxidized *Psa*BCCP (PDB ID: 1EB7) was used to assess redox-related structural changes.

The two mixed-valence structures were identical, and the main observable structural differences were the formation of two small helices (residues 23–26 and 310–313, the latter located at the dimer interface) in the *Ng*BCCP structure, which were not defined in *Psa*BCCP, and the formation of a shorter helix (123–134) marked in red in Figure 5A. These changes occurred in non-conserved regions and can be explained by differences in the amino acid sequence.

Major conformational changes were observed between the two oxidation states, similar to the ones identified between the two oxidation states of the *Psa*BCCP structures [11], *P. pantotrophus* [12] and *N. europaea* [26] BCCPs: the conserved loops in the *Ng*BCCP C-terminal domain, 218–246 loop, and residues 282–290 that extend the alpha helix. These dramatic changes occurred upon reduction of the E heme and were concomitant with further conformational changes in the N-terminal domain (71–118), namely, the *β*-strand between the two heme domains, the alpha helix at the catalytic site (P heme), and the loop (71–81) that contained His75 (P heme distal ligand). Upon reduction, this histidine residue moved to the dimer interface and the catalytic site became 5-coordinated, with an empty coordination for substrate binding.

### 2.4. Dimer Interface

The dimer interface of *Ng*BCCP was analyzed to explain the different properties of its monomer–dimer equilibrium, as this enzyme only dimerizes in the presence of calcium ions and at high protein concentration [5]. In fact, when compared with *Psa*BCCP (a symmetric dimer with an interface surface area of approximately 1585 Å^2^), the residues involved in the dimer interface from the two chains were different in *Ng*BCCP (interface surface areas of 1714 and 1764 Å^2^ in chains A and B), in agreement with the non-crystallographic symmetry of the dimer. Moreover, the interface had no salt bridges, and there were fewer hydrophobic residues, especially in the N-terminal interface, which supported the previous hypothesis that this region is less hydrophobic [5].

One other striking difference of the *Ng*BCCP dimer interface reflected a difference in the amino acid sequence of this enzyme [5] (see Figure S3 in Supplementary Materials). In all the known structures of mixed-valence BCCPs that require reductive activation, there is a π-stacking interaction between Gly72 and Trp73 (*Psa*BCCP numbering) of opposite monomers [11], which stabilizes the loop carrying the P heme distal histidine ligand. This tryptophan residue was not present in *Ng*BCCP, and instead there was a glycine residue, Gly77. However, in the *Ng*BCCP dimer, the 71–81 loop carrying His75 was close to the other monomer, with Gly77C*α* of each monomer at 4.2 Å. This arrangement was stabilized by a hydrogen bond between Asn51-Met69.

### 2.5. The Active Site

**Figure 6:**
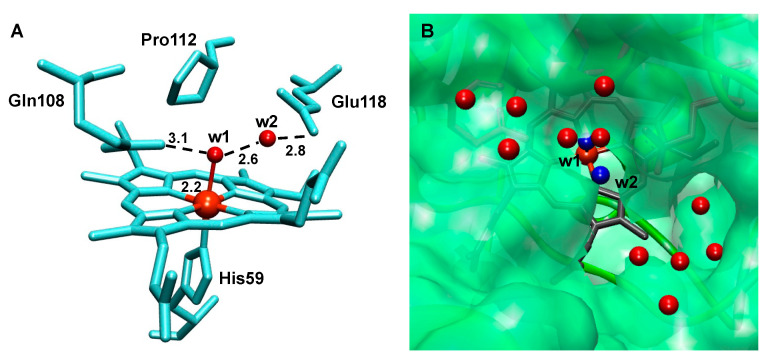
Structure of the P heme active site in chain A. (**A**) In the active site, there are two water molecules, w1 and w2 (small red spheres representing their oxygen atoms), at a short distance from the conserved Gln108 and Glu118 side chains. (**B**) The surface above the P heme pocket showing a channel of waters above w1 and w2 (here as blue spheres). The water molecules around this pocket are represented as red spheres occupying small pockets and groves around the entrance. Figures prepared with BIOVIA Discovery Studio Visualizer 4.5.

Analysis of the electron density maps identified conserved water molecules in the active site of *Ng*BCCP, within H-bonding distances of 2.6–2.8 Å (chains A and B; Figure 6A). One water molecule (w1) was coordinating the P heme iron (Fe-w1 distance of 2.2 Å/2.1 Å), and it was also within a favorable H-bond distance to Gln108. The other water molecule (w2) was close to the P heme iron (4.1 Å) and to Glu118, forming a H-bond (2.8 Å/2.7 Å). This arrangement was not unique, and it was also observed in the P heme cavity of *P. pantotrophus* (2C1V), *N. europaea* (1IQC), and *S. oneidensis* (3O5C) mixed-valence BCCPs. In *Ng*BCCP, there was a direct channel to the P heme, above w2, surrounded by small surface grooves with several water molecules (Figure 6B) that constituted the substrate channel, and these water molecules can also play a role in the proton transfer during catalysis. The P heme cavity was composed of several conserved residues, Phe97, Gln108, Pro112, Glu118, and Met119, with the p*Ka* value of Glu118 predicted to be 6.1–6.4, using the *PropKa*.

### 2.6. The Azide-Inhibited NgBCCP

**Figure 7:**
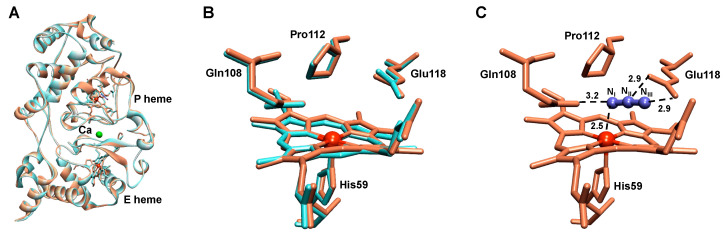
Structure comparison of the *Ng*BCCP active (blue) and azide-inhibited (orange) forms. Overall, the protein fold is conserved (**A**), as well as the residues in the active site (**B**). In the inhibited form there is an azide molecule, H-bonded to the conserved Gln108 and Glu118 (**C**). Figures prepared with BIOVIA Discovery Studio Visualizer 4.5.

The structure of the cyanide-soaked *Ng*BCCP in the active form had a similar fold to the non-inhibited form, with an RMSD of 0.35 Å among all Cα atoms, as observed in the superimposed structures of the monomers and in the structure of the active site (Figure 7A,B). The azide molecule interacted directly with the P heme iron, effectively blocking the active site (Figure 7C). The azide occupied approximately the same position as the two water molecules (w1 and w2), and N_II_ and N_III_ were H-bonded to Glu118 side-chain oxygen atoms, while N_I_ coordinated the ferric heme at 2.5 Å.

## 3. Discussion

### 3.1. NgBCCP Inhibiting Compounds

The binding studies indicated that among the compounds studied, cyanide had the highest affinity for the active site with a *K_app_* of 4 µM, a value that was similar to the one reported for *R. capsulatus* (4 µM) and *P. pantotrophus* (5 µM) BCCPs [19,20], but it was much lower than the affinity reported for *P. aeruginosa* BCCP (23 µM [27]), an indication that the P heme cavity in *Ng*BCCP was more accessible than the one in this latter enzyme.

Cyanide was found to bind only at the P heme, while azide bound preferably to the P heme but also to an allosteric site, as a mixed-inhibition mechanism (non-competitive/partially competitive) was observed. It was reported that azide interacted with the E heme of *N. europaea* BCCP and displaced its coordinating methionine at high concentrations [22], but our visible titrations did not support the binding of azide to the E heme in *Ng*BCCP, and so this allosteric site remains unknown.

Imidazole bound with two distinct modes: at lower concentrations it bound to the P heme, and at higher concentrations it also bound to the ferrous E heme. Analysis of the UV-visible spectra indicated that the E heme methionine S-Fe bond was relatively weak (a temperature-dependent high/low-spin equilibrium was observed [5]); thus, at concentrations above 50 mM imidazole, it was proposed that imidazole competes and dissociates the methionine from the ferrous heme. This effect has been reported for the binding of exogenous ligands to cytochrome *c*_1_, in which it was observed that when imidazole was titrated the usually observed hyperbolic dependence on ligand concentration became more complex [28]. Similar to our proposal for *Ng*BCCP, the authors explained the result as imidazole promoting a conformational change followed by bond formation with the heme iron [28]. We proposed that the binding of imidazole to the reduced E heme changes its reduction potential to lower values (as observed in other heme-binding proteins) [29], and consequently there was oxidation of the E heme, which was consistent with the observed red shift in the Soret band to 410 nm.

Regarding the inhibition of *Ng*BCCP catalytic activity, cyanide was the most efficient inhibitor with a *K_i_* of 0.4 µM, which was similar to the values reported for the BCCPs from *N. europaea* (0.153 µM, pH 7.0) and *P. aeruginosa* (7.1 µM, pH 6.0) [18,22]. However, the free enzyme, in the absence of substrate, showed no observable change in the Soret band of the P heme upon addition of 0.4 µM cyanide. One possible explanation for this low *K_i_* value could be that the enzyme turnover is essential to form a cyanil radical, which is extremely reactive and is formed in the reaction of the cyanide anion (CN^−^) with the oxoferryl intermediate species (Fe^4+^=O, Compound I), possibly leading to an irreversible inhibition of the enzyme at low cyanide concentrations, as proposed for the horseradish CCP [30]. However, even if a similar mechanism for BCCPs might be expected there are no examples in the literature of the mechanism of catalysis in the presence of cyanide for this family of enzymes. Considering that the inhibition mechanism of cyanide is purely competitive, the free enzyme had a high affinity for cyanide, which was of the same order of magnitude as the affinity for the substrate (4 ± 1 µM) [5].

Azide also bound the active site, but its *Ki* value was higher (in the millimolar range). The *K_i_* determined for azide (41 ± 5 mM at pH 7.5) was much higher than the one reported for *P. aeruginosa* BCCP (*K_i_* of 3.1 mM at pH 6.0 [18]), but it was in the same order of magnitude as its binding constant *K_app_*, indicating that inhibition was mainly due to binding of azide to the active site, competing with hydrogen peroxide.

In fact, azide had a higher affinity for the *Ng*BCCP at a lower pH, and this was because of the proportion of HN_3_/N_3_^−^ (p*Ka* of 4.5) and possibly the protonation state at the P heme cavity, as described for yeast CCP [31]. However, at pH 6.0 the enzyme quickly lost activity, and therefore it was not possible to determine a *K_i_* as two effects occurred: inhibition by azide and inhibition due to pH (protonation of key residues, vide infra). Studies of *N. europaea* BCCP using protein film voltammetry also showed a mixed-inhibition by azide at pH 7.0, with a *K_i_* of 4.7 mM and a *K_i_’* of 80 mM [22].

The difference in *K_i_* values between cyanide and azide can be explained by their size, as access through the substrate channel will be hindered for larger molecules. In addition, the azide anion can interact with specific residues at the entrance of the active site or on the surface of the enzyme, changing its conformation and modulating its affinity for the substrate, or different binding modes inside P heme pocket might occur. Nevertheless, in the inhibited structure no other bound azide molecule (or binding modes) besides the one shown in the P heme pocket was observed (Figure 7C).

In the kinetic assays in the presence of imidazole, contrary to what was observed in the binding assays, apparently there was a single effect with no differences between concentrations below and above 50 mM imidazole. This indicated that change in the reduction potential of the E heme upon its binding did not significantly affect the enzyme activity at the concentrations used. One explanation for this is the ability of ABTS^2−^ to directly reduce the P heme, and another is that the methionine might not be significantly displaced at the concentrations used in the kinetic assays (about half of the *K_i_’*), so that intramolecular electron transfer was either not required or not significantly affected. The assays performed at higher concentrations of imidazole showed little activity and could not be addressed by steady-state kinetics.

Overall, the exogenous ligands tested here competed with the substrate for the active site of *Ng*BCCP, inhibiting its catalytic activity. The different modes of binding between the two small molecules, cyanide and azide, and imidazole, can be explained by their size and charge. The small, negatively charged molecules can more easily access the P heme through the substrate channel, while binding of the bulkier neutral imidazole (pH 7.5) seems to be hindered, and the neutral to positively charged surface around the E heme could drive the binding of imidazole to this heme.

### 3.2. NgBCCP Crystal Structure

Here is presented the first structure of an active and inhibited form of a BCCP isolated from a pathogenic bacterium. The detailed analysis of the three-dimensional structure of the *Ng*BCCP active state can explain the different spectroscopic properties and dimer formation of this enzyme. The dimer interface analysis indicated that there were few interactions stabilizing the dimer at the N-terminal domain of this enzyme. For instance, the additional small helix at the C-terminus (310–313) close to the dimer interface was unusual, since in all other BCCPs this region does not retain any significant secondary structure, as predicted for protein–protein interfaces [32]. As a result, only one of the four residues, Arg313, formed a H-bond with the opposite monomer. Furthermore, the Met69 carboxyl group formed a H-bond with Asn83 that coordinated the calcium ion, which suggested that, in the absence of calcium ions, small changes in the protein structure will hamper the formation of the dimer or decrease its stability. In fact, in *P. pantotrophus* BCCP, this loop was already described as being responsible for reducing the area and the hydrophobic character of the dimer interface in the oxidized enzyme [12].

The analysis of the dimer interface suggested that in vitro the soluble *Ng*BCCP was not able to form a stable dimer as there was a weaker interaction and a more dynamic monomer/dimer equilibrium. Nevertheless, in vivo this enzyme did not freely diffuse in the periplasm, and the N-terminal anchor restricted its movements and increased its local concentration at the outer membrane. This high local concentration of *Ng*BCCP at the membrane enabled the enzyme to favorably interact with nearby protein monomers, enabling dimer formation.

In the structure of the active state, the P heme iron atom was distantly coordinated by the oxygen of a water molecule at 2.2 Å. This was in accordance with the previously reported spectroscopic data for the mixed-valence *Ng*BCCP, which at cryogenic temperatures had the EPR signal of a low-spin species, assigned to a 6-coordinated heme with a water molecule as sixth distal ligand [5] (H_2_O/His 6-coordinated P heme). It should be noted that at room temperature the protein structure was dynamic, and the P heme presented features of a high-spin 5-coordinated heme [5] (His 5-coordinated P heme).

As previously described, azide had a higher affinity at a lower pH (*K_app_* of 3.8 mM azide), and as proposed for the yeast azide-inhibited CCP, this could be due to the influence of two ionizable groups [31]. One was the azide itself, HN_3_/N_3_^−^ with a p*Ka* of 4.5, and the other was Glu118 (predicted p*Ka* of 6.1–6.4), which was in close proximity to the azide molecule (2.9 Å). Therefore, at lower pH values, azide was stabilized at the catalytic site by H-bonds. In fact, when designing and choosing plausible inhibiting compounds, it is essential to consider how exogenous ligands bind at different pHs and what the protonation state of each key residue is in the active site at a given pH value. For instance, the pH of the human cervix, the primary site of *N. gonorrhoeae* infection in women, is on average 6.8 in the proliferative state and 6.1 in the secretory stage [33]. Therefore, these new *Ng*BCCP structures are essential in future computational studies to test these pH effects and to improve the affinity by designing compounds with functional groups present in these inhibitors.

### 3.3. The Catalytic Mechanism

Little is known about the catalytic mechanism of BCCPs as most studies have been focused on the catalytic mechanism of eukaryotic cytochrome *c* peroxidases. The generally accepted catalytic mechanism for BCCPs proposes that electrons from a small redox protein (LAz in the case of *Ng*BCCP [7]) are transferred to the E heme, which becomes reduced. Reduction of the E heme leads to conformational changes, more pronounced around the P heme, which loses it distal axial ligand and becomes available for substrate binding—the reductive activation. The electron is then transferred from the E heme to the P heme, where the peroxidatic reaction occurs. This electron transfer is mediated through a “charge-hopping mechanism” through the highly conserved tryptophan residue [34], Trp98 in *Ng*BCCP, positioned in between the propionate groups of the two hemes (Figure 4).

At the active site, two conserved residues, Glu118 and Gln108, are proposed to play an important role in the formation and stabilization of the oxoferryl (Fe^4+^-oxo) intermediate species and Glu118, in particular, in the cleavage of the hydrogen peroxide O-O bond. An identical role was proposed for a conserved glutamate residue in the heme cavity of MauG proteins, which has a similar heme cavity to BCCPs [35,36,37]. The importance of these two residues in catalysis was proven by site-directed mutagenesis studies, which showed that these single residue variants were inactive [20].

**Scheme 2:**
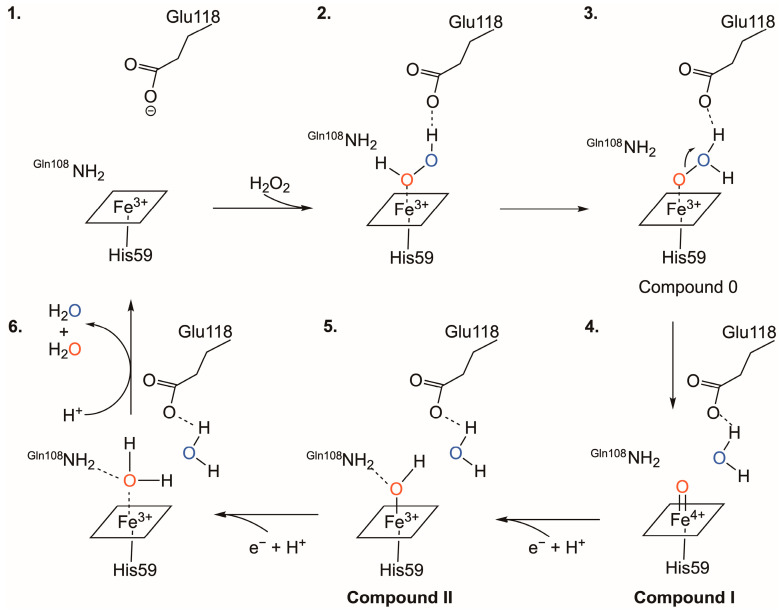
Proposed catalytic mechanism of bacterial peroxidases. (**1.**) P heme is 5-coordinated. (**2.**) In the presence of hydrogen peroxide, there is formation of a peroxide-bound complex, and Glu118 forms a hydrogen bond with one of the oxygens (blue O). (**3.**) The species formed is named Compound 0. (**4.**) The cleavage of O-O bond releases one water, and forms Compound I. (**5.**) Compound I can receive protons from the solvent forming Compound II. (**6.**) Transfer of an additional proton releases a second water molecule.

The comparison between the active site of *Ng*BCCP and that of eukaryotic peroxidases indicated that instead of a histidine residue in the latter, *Ng*BCCP had Glu118. This residue was further away from the water molecule (w1, ≈ 4 Å) than what was observed in those enzymes (≈3.5 Å). However, a mechanism similar to the one proposed for those enzymes was perceived for bacterial peroxidases. In fact, the glutamate’s side chain had a p*Ka* value of ≈4, but this value was different in the heme pocket as it was dependent on the surrounding hydrophobic environment. A p*Ka* of 6.1–6.4 was estimated for Glu118 using bioinformatic tools based on the active structure of *Ng*BCCP. A p*Ka* with a similar value was estimated experimentally in the steady-state kinetic assays using ABTS^2−^ as electron donor [5] (p*Ka_1_* of 5.9), which can thus be tentatively assigned to Glu118 and needs to be unprotonated for optimum activity (Scheme 2). The other conserved residue in the P heme cavity was Gln108, which, although it does not have an ionizable side-chain, was H-bonded to other residues in the heme pocket and is proposed to form a H-bond to the oxygen in Compound I. The other p*Ka*_2_ value of 8.4 ± 0.1, estimated in the same kinetic assays [5], could be assigned to solvent water molecules in the heme pocket.

Therefore, a catalytic mechanism was proposed based on the structural data presented here, knowledge about BCCP catalytic intermediate species, and p*Ka* values estimated experimentally in the kinetic assays and bioinformatically using the three-dimensional structure of *Ng*BCCP (Scheme 2). In this mechanism, hydrogen peroxide enters the catalytic cavity and one of its oxygen atoms forms a H-bond with Glu118 (the position that in the absence of substrate is occupied by the oxygen of w2 in Figure 6A), helping to elongate the O-O bond and leading to its cleavage (Scheme 2, state 2–4). Compound I is formed with the release of the first water molecule (Scheme 2, state 4). Compound I is an oxoferryl species, non-protonated as shown in other peroxidases [38]. Proton transfer from a solvent-water molecule aided by Gln108 (positioned at 3.1 Å from w1 in Figure 6A) will form Compound II. Addition of another proton releases a second water molecule (Scheme 2, state 5–6).

As proposed in the schematic mechanism (Scheme 2), the structural configuration of the mixed-valence *Ng*BCCP active site (Figure 7A) is similar to the final step of the catalytic cycle (Scheme 2, state 6) and can thus be regarded as an intermediate species.

Other intermediate species proposed in the catalytic cycle of *P. aeruginosa* [34] and *N. europaea* [22,39] BCCPs are protein radicals, namely, the tryptophan radical (Trp98 between the two hemes) and the porphyrin radical, respectively. Their role in the catalytic cycle is not completely understood. It is possible that these protein radicals are essential for fast long-range electron transfer between the E heme and the P heme. In fact, the catalytic mechanism of *N. europaea* is proposed to be different from the classical BCCPs as this enzyme does not require reductive activation. The major difference according to the authors is that the E heme does not store the electron for the catalytic reaction in the P heme, as the hydrogen peroxide binds the oxidized enzyme forming the porphyrin π-cation radical without requiring a reduced E heme [22,39]. Nevertheless, a recent comparative electrochemical study with *N. europaea* and *Shewanella oneidensis* BCCPs demonstrated that a *S. oneidensis* BCCP H81G mutant (histidine residue next to the P heme distal histidine ligand His80) shows the same electron stoichiometry and pH dependence as the *N. europaea* BCCP [40]. Therefore, the intermediate species in the BCCP catalytic mechanism might be the same, even if *N. europaea* BCCP does not require reductive activation.

As a final remark, it would be interesting to compare the active sites of different eukaryotic CCPs and their proposed catalytic mechanisms, since BCCPs have a highly conserved active site. The horseradish peroxidase (HRP) and ascorbate peroxidase (APX) have similar active sites but with a histidine as acid-base catalyst [41] instead of the glutamate in BCCPs. Quantum mechanical/molecular mechanical simulations of HRP suggested a mechanism similar to the one proposed in Scheme 2, in which the histidine is not protonated and the protons that form the first water molecule are from the hydrogen peroxide. A variation of this mechanism suggests that the O-O cleavage occurs with the assistance of a nearby “catalytic” water molecule [42]. Other features observed in eukaryotic CCPs, such as the movement of heme pocket residues during catalysis, described for Arg48 in yeast CCP, which moves toward the oxoferryl species in the Compound I structure [43], cannot be assessed without a BCCP structure previously incubated with hydrogen peroxide. However, to trap this intermediary species, this must be performed with BCCP mutants [34,44] or with a BCCP such as the one isolated from *N. europaea*, which has a 5-coordinated P heme iron in the full oxidized state.

## 4. Materials and Methods

### 4.1. NgBCCP Production and Purification

*Ng*BCCP was produced and purified as described by Nóbrega et al. [5]. In sum, the globular domain of *Ng*BCCP was cloned into pET22b (+) plasmid, which adds a N-terminal signal peptide (*pelB*), to direct the protein to *Escherichia coli*’s periplasm and a C-terminal His-tag. This recombinant protein was heterologously produced in *E. coli* BL21 (DE3) strain (Novagen) co-transformed with the pET22b-*NgBCCP* and pEC86, which encodes all the machinery for *c-*type heme biosynthesis and maturation. *Ng*BCCP was purified in two chromatographic steps. In the first chromatographic step the periplasmic extract was loaded onto a HisTrap 5 mL column (Cytiva, Marlborough, US) followed by size-exclusion chromatography using a Superdex 200 16/600 (Cytiva, Marlborough, US). The purity of the protein sample was assessed by SDS-PAGE and by the absorption ratio A_402 nm_/A_280 nm_. The final sample was stored at −80 °C until further use.

### 4.2. UV-Visible Spectroscopy

For the *Ng*BCCP inhibition studies, the UV-visible spectra of 2 µM mixed-valence *Ng*BCCP solutions (in 10 mM HEPES pH 7.5, 1 mM sodium ascorbate, 5 µM diaminodurol, 1 mM CaCl_2_) were recorded in the presence of increasing concentrations of inhibitors: cyanide (0–95 µM), azide (0–225 mM), and imidazole (0–190 mM) sodium salts prepared in Milli-Q water (imidazole pH was adjusted to 7.5). The difference in the Soret band at specific wavelengths (ΔA) was plotted as a function of inhibitor concentration *i* and data were fitted with the following binding model (Equation (1)) for one single binding site, where B_max_ is the maximum difference at which all protein molecules are bound to the inhibitor and *K_app_* is the apparent dissociation constant:

(1) $$∆A=\frac{B_{max}.i}{K_{app}+i}$$

### 4.3. Steady-State Kinetics with Inhibitors

The activity of *Ng*BCCP using 2,2′-azino-di-(3-ethyl-benzothiazoline-6-sulphonic acid (ABTS^2−^, Sigma, from Merck KGaA, Darmstadt, Germany) as electron donor was determined by monitoring the increase in absorbance at 420 nm (ε_420 nm_ = 36 mM^−1^ cm^−1^) over time as a result of ABTS^2−^ oxidation [45] in the presence of substrate (H_2_O_2_) in an Agilent Diode Array. The assay was performed at 25 °C and the reaction was initiated with 10 nM pre-activated *Ng*BCCP, added to a reaction mixture containing 10 mM HEPES pH 7.5, 10 mM NaCl, 1 mM CaCl_2_, 3 mM ABTS^2−^, inhibitor, and H_2_O_2_. *Ng*BCCP was pre-activated for all kinetic assays in 5 μM enzyme stocks in 10 mM HEPES pH 7.5, 10 mM NaCl, 0.2 mM sodium ascorbate, 5 μM DAD, and 1 mM CaCl_2_ during 30 min at room temperature and then used directly in the assay [5].

Various concentrations of cyanide (0–70 µM), azide (0–100 mM), and imidazole (0–170 mM) sodium salts were tested at three H_2_O_2_ concentrations: 10, 25, and 100 µM. The initial rates of these assays were determined and used to estimate *K_M.app_* and *V_M.app_* for each concentration of the inhibitor, using different linearizations.

The *K_i_* values for competitive inhibition (2) and mixed-inhibition (3) were determined according to the following equations:

(2) KM.app=KM1+iKi

(3) VM.app=Vmax1+iαKi

where *K_M,app_* and *V_M,app_* are determined from the Michaelis–Menten equation at each concentration *i* of the inhibitor. *V_M_* and *K_M_* are the values determined without any inhibitor and α was estimated from Dixon and Cornish–Bowden plots, which give apparent *K_i_* and *K_i_*’ since *K_i_*’ = *αK_i_*. Although the Cornish–Bowden plot gives a good estimate of *K_i_*, the above equations were used to calculate the *K_i_* for each inhibitor concentration, separately. The final reported *K_i_* is an average of these values.

### 4.4. Crystallization of NgBCCP in the Mixed-Valence State

As-isolated *Ng*BCCP (oxidized state) was used for extensive screening of crystallization conditions by vapor diffusion methods. Droplets consisted of 0.7 µL protein solution (10 or 20 mg mL^−1^ in 20 mM Hepes pH 7.5) and 0.7 µL precipitant solution, equilibrated against 500 µL of the same precipitant solution in the reservoir. In some of the assays *Ng*BCCP was also pre-incubated with 2 mM CaCl_2_. Two preliminary conditions using Jeffamine SD2001 or pentaerythritol propoxylate (5/4 PO/OH) as precipitant were found, the former at 20 °C and the latter at 4 °C.

When Jeffamine was used as precipitant, several crystals of as-isolated *Ng*BCCP, pre-incubated with 2 mM CaCl_2_, were obtained in 0.1 M MES pH 5.5, Jeffamine SD2001 (25–27%), and 0.1 M sodium malonate at 4 °C. This condition was further optimized by screening various additives to reduce crystal multiplicity. The addition of 30% (*v*/*v*) 1,6-hexanediol or 0.1 M hexamine cobalt (III) trichloride to 4 µL drops (ratio of 5:4:1 for protein:reservoir:additive solution) resulted in single crystals.

In the case of 5/4 PO/OH as precipitant, crystals of as-isolated NgBCCP, pre-incubated with calcium chloride, were obtained in 0.1 M MES, pH 6.0 or 6.5, 5/4 PO/OH (25–30%) with or without 0.2 M NaCl, at 20 °C.

Crystallization of *Ng*BCCP in the mixed-valence state was performed using the conditions established before for the as-isolated enzyme, using sodium ascorbate and a mediator (flavin mononucleotide, FMN) to reduce the E heme, under an anoxic environment. The best crystals were obtained in 30% 5/4 PO/OH and 0.1 M MES pH 6.0 in the presence of 2 mM CaCl_2_, 10 mM sodium ascorbate, and 0.2 mM FMN using a 20 mg mL^−1^ protein solution previously incubated with calcium chloride, sodium ascorbate, and FMN in the same concentrations as the reservoir solution. These drops (4 µL final volume, 1:1 ratio of protein:reservoir, 500 µL reservoir solution in a 24-well plate) were prepared in a Coy Lab anaerobic chamber (2% hydrogen, 98% nitrogen atmosphere) using the sitting drop vapor-diffusion method and kept in an incubator at 30 °C. After seven days, crystallization plates were removed from the anaerobic chamber to 20 °C for crystal flash-cooling in liquid nitrogen. For cryopreservation, the harvesting solution (0.1 M MES pH 6.0 and 30% 5/4 PO/OH) was supplemented with 20% glycerol.

The crystals of the inhibited form were obtained by soaking the crystals of mixed-valence *Ng*BCCP in a harvesting solution in the presence of 10 mM sodium azide during 30 min, prior to flash-cooling. Soaking with other inhibitors, sodium cyanide and sodium fluoride, added in similar concentrations, was also attempted, though unsuccessful as the crystals were severely damaged.

### 4.5. Data Collection and Processing

X-ray diffraction data were collected to completeness on an in-house X-ray diffractometer (IμS 3.0 microfocus D8 Venture with copper Kα radiation), coupled to a CMOS Photon 100 detector at 110 K from a *Ng*BCCP crystal in the mixed-valence active state. This crystal diffracted to a maximum resolution of 1.8 Å. Intensities were processed, integrated, and scaled using PROTEUM3 software pipeline (version 2016.2; Bruker AXS Inc.) and converted to observed structure factors using SCALEPACK2MTZ and TRUNCATE from the CCP4 suite [46]. The crystals belonged to space group *P*2_1_2_1_2_1_, with unit-cell parameters *a* = 78.9 Å, *b* = 88.8 Å, and *c* = 93.1 Å. The asymmetric unit comprised one dimer of *Ng*BCCP with a solvent content of 43%.

Another crystal grown under similar conditions was cryo-cooled by adding 20% (*v*/*v*) glycerol to the harvesting solution and stored in liquid nitrogen. X-ray diffraction data from this crystal were collected to 1.4 Å resolution at the Swiss Light Source (SLS, beamline X06DA PXIII) using radiation of 1 Å wavelength. This crystal contained 41% solvent, also considering a dimer of *Ng*BCCP in the asymmetric unit of the *P*2_1_2_1_2_1_ space group. Unit cell parameters were *a* = 78.8 Å, *b* = 89.1 Å, and *c* = 89.7 Å. X-ray diffraction data from the azide-soaked crystal were collected at ESRF (beamline BM30, Grenoble), using radiation of 0.979 Å wavelength. The crystal diffracted up to 2.3 Å resolution, with unit cell parameters *a* = 79.1 Å, *b* = 89.1 Å, and *c* = 94.8 Å, containing one dimer in the asymmetric unit (45% solvent content) in space group *P*2_1_2_1_2_1_. All synchrotron data were integrated with program MOSFLM [47] and scaled with AIMLESS [48] from the CCP4 suite. Data collection and processing statistics are presented in Table 1.

### 4.6. Structure Solution and Refinement of Preliminary Model

A solution to the phase problem was obtained by molecular replacement, using as observed structure factors the in-house-collected 1.8 Å resolution data and as the search model the monomer of the known structure of bacterial cytochrome *c* peroxidase from *P. aeruginosa* (PDB ID 2VHD). This model showed 48% amino acid sequence identity to *Ng*BCCP. The search was performed for all space group choices in the working point group and for two copies of the search model in the asymmetric unit. A molecular replacement solution was found in space group *P*2_1_2_1_2_1_ using program PHASER [49] implemented in PHENIX [50], with a log-likelihood gain of 352.23 and a TFZ of 21.6. Phenix.autobuild was used for model improvement in the calculated electron density, building 2 monomers of *Ng*BCCP with a total of 648 amino acid residues and 535 water molecules. After model building and refinement, the Rwork and Rfree were 21.9% and 25.5%, respectively. This model was used as phases to obtain the structures of mixed-valence *Ng*BCCP active state, at 1.4 Å, and azide-inhibited, at 2.3 Å resolution. Model building and refinement of all three structures was carried out in iterative steps using COOT [51] and REFMAC5 [52]. Final rounds of refinement with PDB_REDO [53] produced final structures with Rwork and Rfree values of 19.6% and 22.0% for the 1.4 Å resolution *Ng*BCCP active structure and 18.0% and 23.3% for the 2.3 Å resolution *Ng*BCCP azide-inhibited structure. For all 3D structures, the residues have backbone and angles in the allowed region of the Ramachandran plot, with 96% in the favored region, except for outlier residues Phe97, Glu118 in the P-heme binding pocket, and Phe265 in the E heme binding pocket. All data collection and refinement statistics are summarized in Table 1.

### 4.7. Protein Structure and Surface Analysis

All structures were analyzed, and their images prepared on BIOVIA Discovery Studio Visualizer 4.5 except when otherwise stated in the text. PDBeFold was used to compare protein structures and determine the root mean square deviation (RMSD) and other parameters (https://www.ebi.ac.uk/msd-srv/ssm/cgi-bin/ssmserver (accessed on 2 February 2023)) [54]. PDB files of superimposed crystal structures were created in Chimera 1.10.2. The dimer interfaces were analyzed in PDBePISA (‘Protein interfaces, surfaces and assemblies’ service PISA at the European Bioinformatics Institute; http://www.ebi.ac.uk/pdbe/prot_int/pistart.html (accessed on 21 November 2022)) [55], which gives information about which residues compose the interface, the type of interactions formed, and its symmetry.

The protein electrostatic surface was analyzed using Adaptive Poisson–Boltzmann Solver (APBS [56,57]). The PDB files were treated in PDB2PQR [58] (https://server.poissonboltzmann.org/pdb2pqr (accessed on 23 November 2022)) which applies a forcefield (AMBER), assigns charges and radius parameters, optimizes hydrogen bonding networks, and assigns protonation states according to pH (7.0) using PROPKA [59]. The final PQR file from PDB2PQR was used as input in the APBS (http://www.poissonboltzmann.org/ (accessed on 23 November 2022)) to determine electrostatic properties using default parameters. The solvent accessible surface was colored according to electrostatic potential in Chimera, from –5 to +5 kT/e (red to blue).

PropKa (https://www.ddl.unimi.it/vegaol/propka.htm (accessed on 2 February 2023)) [59] was also used to predict the p*Ka* values using the coordinates from the crystal structure as input.

## 5. Conclusions

*Ng*BCCP is a highly conserved enzyme in *N. gonorrhoeae* strains, anchored to the outer membrane. This dihemic enzyme, unlike other classical BCCPs, only dimerizes at high protein concentration and in the presence of added calcium ions. This can be explained by the *Ng*BCCP dimer interface, which, in comparison to other classical BCCPs, is less hydrophobic and has fewer inter-subunit interactions in its N-terminal domain. Its location in vivo, anchored to the outer membrane, and the interface properties suggest that the dimer assembly does not need to be as strong as for other enzymes that freely diffuse in the periplasm.

One unique spectroscopic feature of the *Ng*BCCP P heme is that it remains partially as a high-spin species at cryogenic temperatures. This reflects changes in the surroundings of the P heme cavity and calcium site that affect the p*Ka* of the solvent-derived molecule coordinating P heme iron, although comparison of the available BCCP structures showed no significant differences. A dynamic monomer–dimer equilibrium could cause significant changes at the P heme, namely, in the conserved loop region, carrying the P heme distal histidine ligand. In fact, this loop is not stabilized at the dimer interface by the conserved tryptophan *π*-stacking motif between monomers found in other classical BCCPs.

The P heme cavity is accessible to its substrate but also to other exogenous ligands, specifically cyanide, which has high affinity for the active site. Furthermore, the binding of cyanide, imidazole, and azide inhibits *Ng*BCCP catalytic activity mainly by competitive inhibition, as they bind directly to the P heme. This was demonstrated for the first time in an azide-inhibited BCCP structure in which the active site was blocked by this ligand and therefore prevented substrate binding.

The proposed catalytic mechanism based on the catalytic intermediate species and on the kinetic data suggests that the two waters in the P heme pocket of the *Ng*BCCP structure can be part of an intermediate step of the catalytic cycle.

*Ng*BCCP is conserved in *N. gonorrhoeae*, a human pathogenic bacterium, but absent in human cells and accessible in the outer membrane, making this enzyme a good candidate for immunization. Thus, the analysis of these structural data and further studies with additional exogenous ligands and/or compounds with functional groups present in the inhibitors studied here should be pursued to develop new drugs with higher affinity and specificity.

## Figures and Tables

**Table 1:** Statistics of data collection, processing, model building and refinement.

	*Ng*BCCP (Active, 1.8 Å)	*Ng*BCCP (Active, 1.4 Å)	*Ng*BCCP (Azide-Inhibited, 2.3 Å)
*Data Collection and Processing*			
Source	IμS 3.0	PXIII (X06DA), SLS	BM30, ESRF
Detector	CMOS Photon 100	PILATUS 2M-F	ADSC Q315r CCD
Wavelength (Å)	1.5418	1.0	0.979
*a* (Å)	78.9	78.8	79.1
*b* (Å)	88.8	89.1	89.1
*c* (Å)	93.1	89.7	94.8
Space group	*P*2_1_2_1_2_1_	*P*2_1_2_1_2_1_	*P*2_1_2_1_2_1_
Molecules per ASU	2	2	2
Matthews coefficient (Å^3^.Da^−1^)	2.17	2.10	2.23
Resolution range (Å)	23.81–1.80 (1.85–1.80)	39.90–1.40 (1.42–1.40)	65.00–2.30 (2.38–2.30)
<I/σI>	10.3 (2.31)	11.4 (2.1)	9.2 (2.2)
Wilson B-factor	14.5	12.7	27.4
R*_merge_* (%) *	15.7 (62.6)	6.8 (65.6)	11.7 (72.3)
R*_pim_* (%) ^†^	-	4.2 (44.9)	8.0 (48.7)
Half-dataset correlation CC1/2	-	0.999 (0.813)	0.995 (0.762)
Multiplicity	10 (7.8)	6.4 (5.6)	5.6 (5.6)
No. of observed reflections	588,364	797,508 (33,769)	169,921 (16,295)
No. of unique reflections	61,589	124,619 (6083)	30,270 (2907)
Completeness (%)	98.8 (99.9)	99.9 (99.3)	97.7 (94.9)
*Model Building and Refinement*			
No. of protein atoms	5080	5096	5032
No. of water molecules	535	577	191
R_work_^‡^ (%)	21.9	19.6	18.0
R_free_^§^ (%)	25.5	22.1	23.3
R.m.s.d. bond lengths (Å)	0.029	0.012	0.015
R.m.s.d. bond angles (⁰)	2.810	1.972	2.124
Average B-factor (Å^2^)			
Protein			
Main-chain (A, B)	20.1, 19.2	17.3, 16.3	37.8, 30.5
Side-chain (A, B)	22.5, 21.4	20.6, 19.5	40.3, 33.5
Heme groups P heme_A, E heme_A P heme_B, E heme_B	12.1, 14.9, 13.0, 13.7	11.0, 15.0 11.0, 12.0	25.2, 36.8 22.9, 21.1
Iron atoms P heme_A, E heme_A P heme_B, E heme_B	13.0, 16.5, 13.9, 15.1	11.5, 14.8 11.2, 12.4	24.2, 36.7 21.9, 24.3
Calcium ions (A, B)	11.7, 10.5	12.8, 10.4	28.3, 22.1
Water molecules	25.9 (535)	26.6 (577)	30.1 (191)
Azide ions (A, B)	-	-	39.8, 51.3
Ramachandran plot			
Residues in favored regions (%)	96.1	96.8	96.1
Residues in allowed regions (%)	2.9	2.3	2.8
Residues outliers (%)	0.9	0.9	1.1
PDB accession codes	6FU3	7ZS8	6QKN

Statistics for the highest-resolution shell are shown in parentheses. * $R_{merge}=\frac{\sum_{hkl}\sum_{i=1}^{n}\left|I_{i}\left(hkl\right)-\overset{-}{I}\left(hkl\right)\right|}{\sum_{hkl}\sum_{i=1}^{n}I_{i}\left(hkl\right)}$, where I is the observed intensity, and $\overset{-}{I}$ is the statistically weighted average intensity of multiple observations. ^†^ $R_{p.i.m.}=\frac{\sum_{hkl}\sqrt{1/\left(n-1\right)}\sum_{i=1}^{n}\left|I_{i}\left(hkl\right)-\overset{-}{I}\left(hkl\right)\right|}{\sum_{hkl}\sum_{i=1}^{n}I_{i}\left(hkl\right)}$, a redundancy-independent version of $R_{merge}$. ^‡^ $R_{work}=\frac{\sum_{hkl}\left|\left|F_{obs}\left(hkl\right)\right|-\left|F_{calc}\left(hkl\right)\right|\right|}{\sum_{hkl}\left|F_{obs}\left(hkl\right)\right|}$, where $\left|F_{calc}\right|$ and $\left|F_{obs}\right|$ are the calculated and observed structure factor amplitudes, respectively. ^§^ *R_free_* is calculated for a randomly chosen 5% of the reflections.

## Data Availability

All protein coordinates reported here are available from the RCSB Protein Data Bank (https://www.rcsb.org/ (accessed on 8 February 2023)).

## Supplementary Materials

The following supporting information can be downloaded at: https://www.mdpi.com/article/10.3390/ijms24076246/s1.
