# Supplementary material for: Structural Characterization of Neisseria gonorrhoeae Bacterial Peroxidase—Insights into the Catalytic Cycle of Bacterial Peroxidases
Source: Int J Mol Sci. 2023 Mar 26;24(7):6246. doi: 10.3390/ijms24076246 (PMC10094543; doi:10.3390/ijms24076246)
Supplement: Supplementary file 1 [file ijms-24-06246-s001.zip › ijms-2240170-supplementary.pdf]

Supplementary Materials

Structural characterization of Neisseria gonorrhoeae bacterial peroxidase – Insights into the catalytic cycle of bacterial peroxidases

Cláudia S. Nóbrega[1,3], Ana Luísa Carvalho[2,3], Maria João Romão[2,3], Sofia R. Pauleta[1,3,*]

[1] Microbial Stress Lab, UCIBIO – Applied Molecular Biosciences Unit, Department of Chemistry, NOVA School of Science and Technology, Universidade NOVA de Lisboa, 2829-516 Caparica, Portugal.

[2] Macromolecular Crystallography Lab, UCIBIO – Applied Molecular Biosciences Unit, Department of Chemistry, NOVA School of Science and Technology, Universidade NOVA de Lisboa, 2829-516 Caparica, Portugal.

[3] Associate Laboratory i4HB - Institute for Health and Bioeconomy, NOVA School of Science and Technology, Universidade NOVA de Lisboa, 2829-516 Caparica, Portugal.

*Correspondence: sofia.pauleta@fct.unl.pt

INDEX

S1. Binding of azide at pH 6.0 p.S2

S2. Mechanism of inhibition p. S2

S3. Crystallographic structures of bacterial cytochrome *c* peroxidases p. S4

S4. Sequence alignment of di-heme bacterial peroxidases p. S5

S1. Binding of azide at pH 6.0

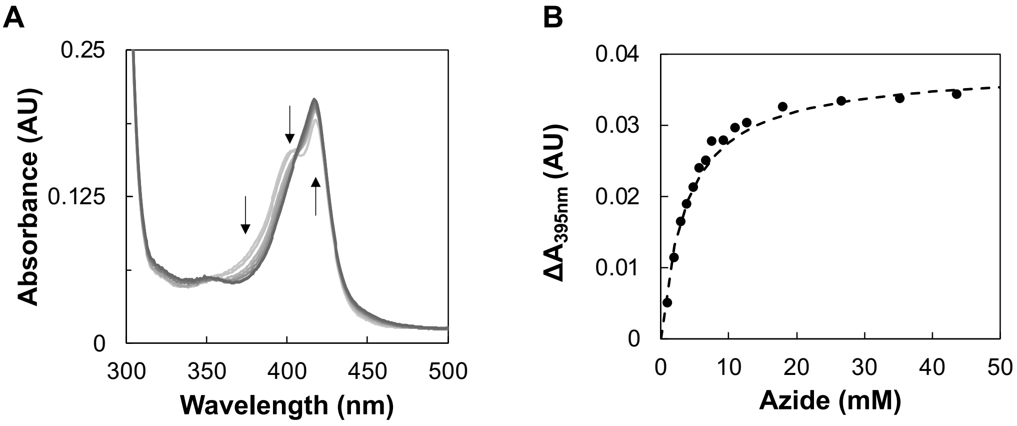

Figure S1. UV-visible spectra of mixed-valence *Ng*BCCP at pH 6.0 in the presence of increasing concentrations of azide (A, B). The arrows indicate the direction of changes in the spectra from the light gray (no inhibitor) to dark gray (maximum inhibitor concentration). The absorption difference at specific wavelength was plotted as a function of inhibitor concentration in solution (B). The dashed line in the binding of azide was fitted with a single binding site equation (see manuscript) with a K_{app} of 3.8 mM.

S2. Mechanism of inhibition

Table S1 – Steady-state kinetics parameters determined for each concentration of cyanide (CN^-), azide (N_3^-) and imidazole (Im).

Cyanide				Azide				Imidazole			
$[CN^-]$ (μM)	K_{M}app (μM)	V_{max} app (μM.s^{-1})	*Ki* (μM)	$[N_3^-]$ (mM)	K_{M}app (μM)	V_{max} app (μM.s^{-1})	*Ki* (μM)	[Im] (mM)	K_{M}app (μM)	V_{max} app (μM.s^{-1})	*Ki* (μM)
0	3.6±0.3	0.74±0.003	-	0	3.6±0.3	0.74±0.003	-	0	3.9±0.3	0.72±0.01	-
10	15.5±0.9	0.67±0.02	0.5±0.3	10	2.5±0.1	0.71±0.001	36.6±2.1	20	3.7±0.4	0.65±0.01	7.6±1.2
20	34.1±10.0	0.74±0.12	0.4±0.3	40	4.4±1.1	0.66±0.010	55.4±3.3	35	3.5±1.2	0.63±0.04	8.6±1.5
35	61.7±9.6	0.77±0.08	0.4±0.3	60	3.8±0.1	0.59±0.001	36.2±2.1	50	3.1±0.9	0.58±0.02	6.4±1.1
50	79.4±45.6	0.75±0.31	0.5±0.4	80	4.5±0.3	0.56±0.003	39.7±2.3	80	4.8±0.02	0.55±0.01	9.1±1.5
70	161.7±33.5	1.0±0.2	0.3±0.2	100	5.6±0.5	0.53±0.004	38.7±2.2	110	6.1±0.3	0.49±0.01	8.6±1.4
								140	7.5±0.3	0.48±0.01	9.8±1.6
								170	9.5±1.6	0.44±0.02	10.3±1.7

Besides the analysis presented in the manuscript, kinetic data were fitted using the non-linear least square method, using the tool Solver in Excel. Using this method, three equations were fitted simultaneously to obtain the parameters, α, β, and K_I, and identity the mechanism of inhibition of each compound (Figure S1 and Table S2). This model only assumes the steady-state conditions and rapid equilibrium of the different forms of the enzyme, with no other approximation. Equations used in the fitting, in which 0 denotes the initial concentration present in the assays, $K'_M = \alpha Ks$, and the general Webb model are the following. K'_M, V'_M and V_M corresponds to K_{Mapp}, $V_{max\,app}$ and V_{max} in the manuscript, respectively.

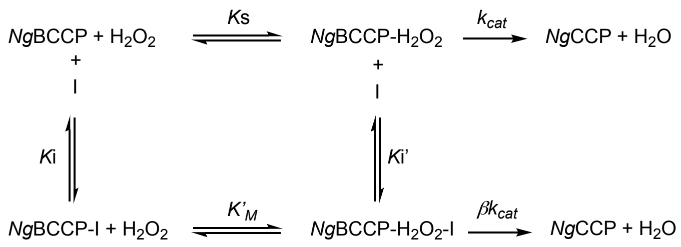

$$\frac{V_M}{V'_M} = \frac{1 + \frac{[I]_0}{\alpha K_I}}{1 + \beta \frac{[I]_0}{\alpha K_I}} \qquad \frac{K'_M}{K_s} = \frac{1 + \frac{[I]_0}{K_I}}{1 + \frac{[I]_0}{\alpha K_I}} \qquad \frac{K'_M}{K_s}\frac{V_M}{V'_M} = \frac{1 + \frac{[I]_0}{K_I}}{1 + \beta \frac{[I]_0}{\alpha K_I}}$$

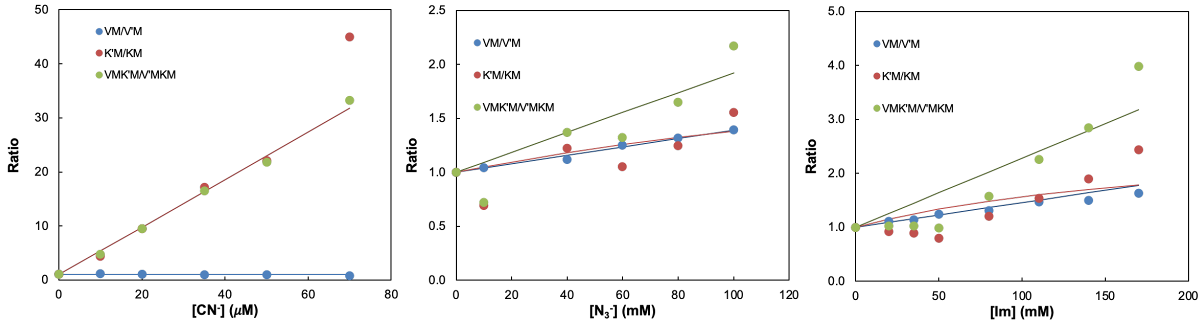

Figure S2 – Representation of the three rations (VM/V'M, K'M/KM, VMK'M/(V'M/KM)) as a function of inhibitor. Experimental data is represented as circles and lines is the fitting. The data obtained from the fitting is presented in Table S2.

The values obtained are presented in Table S2.

Table S2 – Inhibition parameter determined for cyanide, azide and imidazole.

PARAMETER	CYANIDE	AZIDE	IMIDAZOLE
α	1.7 x 10^7	2.4	2.8
β	0.0	0.0	0.0
Ki	2.3 μM	108 mM	78 mM

S3. Crystallographic structures of classical bacterial peroxidases

Table S3- Comparison of BCCP structures in the oxidized (Ox) and mixed-valence (MV) forms. RMSD was determined for the aligned Cα atoms of the superimposed structures in PDBeFold [54]. Z-score and % of sequence identity (%seq), percentage of matched SSEs (%sse – fraction of secondary structure of query identity in the target).

Organism, redox state	PDB ID	Resolution (Å)	RMSD (Å)	Z-score	%seq	Query %sse
Neisseria gonorrhoeae, MV	7ZS8	1.4	-			
Pseudomonas aeruginosa, MV	2VHD	2.3	1.16	19.7	50	84
Pseudomonas aeruginosa, Ox	1EB7	2.4	1.39	14.5	45	60
Nitrosomonas europaea, Ox	1IQC	1.8	1.30	17.0	45	72
Paracoccus pantotrophus, MV	2C1V	1.2	1.28	19.0	45	80
Paracoccus pantotrophus, Ox	2C1U	1.9	1.45	14.9	44	60
Rhodobacter capsulatus, Ox	1ZZH	2.7	1.53	13.4	43	56
Geobacter sulfurreducens MacA, MV	4AAM	2.2	1.08	17.8	47	72
Geobacter sulfurreducens CcpA, Ox	3HQ6	2.0	1.44	14.5	42	64
Shewanella oneidensis, MV	3O5C	1.8	1.16	17.0	45	68
Marinobacter nauticus, OUT/MV	1RZ5	2.4	1.74	13.3	43	60
Marinobacter nauticus, IN/Ox	1RZ6	2.2	1.80	13.0	43	64

S4. Sequence alignment of classical Bacterial Peroxidases

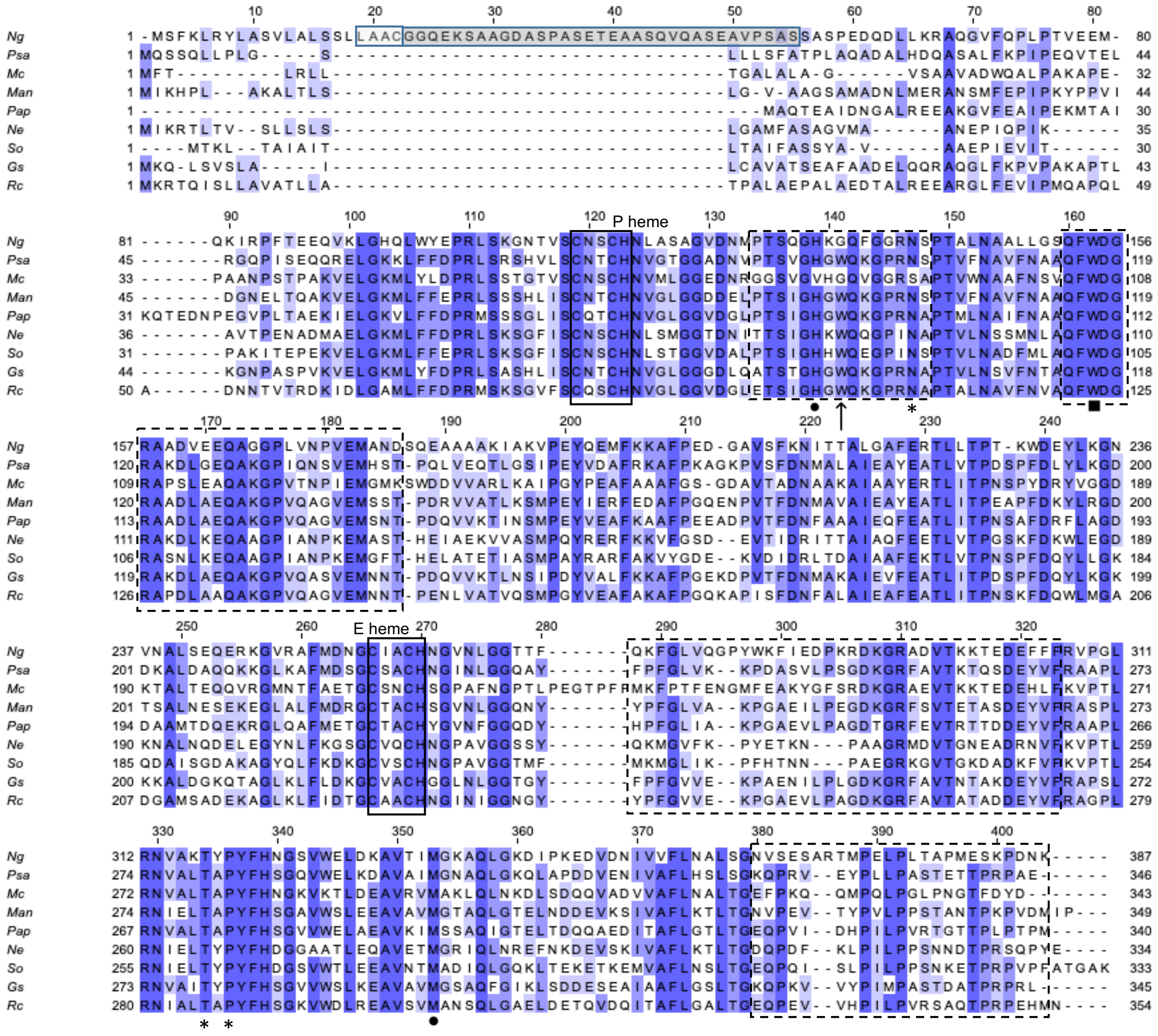

Figure S3 - Multiple sequence alignment of bacterial cytochrome *c* peroxidases primary sequence. *N. gonorrhoeae* FA 1090 (*Ng*; WP_003705279.1), *M. capsulatus* (*Mc*; WP_010959706.1), *Shewanella oneidensis* (*So*; WP_011072214.1), *P. aeruginosa* (*Psa*; WP_003094815.1), *N. europaea* (*Ne*; WP_011111893.1), *Marinobacter nauticus* (*Man*; WP_014420293.1), *Paracoccus pantotrophus* (*Pap*; WP_051419533.1), *Rhodobacter capsulatus* (*Rc*; WP_055209429.1) and *Geobacter sulfurreducens* (*Gs*; WP_010943439.1). The coloring is in accordance with percentage of identity at each position, from darker color box (100 % identity) to white box (≤ 20 % identity). The sequences are sorted by pairwise identity to *Ng*BCCP. The outlined light grey box marks the *Ng*BCCP peptidase II cutting site and the grey box the low complexity region with imperfect sequence repeats, only present in this protein. The loops and flexible regions involved in reductive activation are outlined by dashed black boxes. Some residues are marked as: (●) axial ligands, (*) calcium binding residues, (■) tryptophan at the domain interface, and G77 in *Ng*BCCP highlighted by an arrow.
